# Pin1 inhibition exerts potent activity against acute myeloid leukemia through blocking multiple cancer-driving pathways

**DOI:** 10.1186/s13045-018-0611-7

**Published:** 2018-05-30

**Authors:** Xiaolan Lian, Yu-Min Lin, Shingo Kozono, Megan K. Herbert, Xin Li, Xiaohong Yuan, Jiangrui Guo, Yafei Guo, Min Tang, Jia Lin, Yiping Huang, Bixin Wang, Chenxi Qiu, Cheng-Yu Tsai, Jane Xie, Ziang Jeff Cao, Yong Wu, Hekun Liu, Xiaozhen Zhou, Kunping Lu, Yuanzhong Chen

**Affiliations:** 1 Fujian Institute of Hematology, Fujian Provincial Key Laboratory on Hematology, Fujian Medical University Union Hospital, Fuzhou, 350001 Fujian China; 2 Division of Translational Therapeutics, Department of Medicine and Cancer Research Institute, Beth Israel Deaconess Medical Center, Harvard Medical School, Boston, MA 02215 USA; 3 Fujian Key Laboratory for Translational Research in Cancer and Neurodegenerative Diseases, Institute for Translational Medicine, Fujian Medical University, Fuzhou, 350108 Fujian China

**Keywords:** Acute myeloid leukemia (AML), Pin1 inhibitor, All-*trans* retinoic acid (ATRA), Oncogenic signaling, Leukemia treatment

## Abstract

**Background:**

The increasing genomic complexity of acute myeloid leukemia (AML), the most common form of acute leukemia, poses a major challenge to its therapy. To identify potent therapeutic targets with the ability to block multiple cancer-driving pathways is thus imperative. The unique peptidyl-prolyl cis-trans isomerase Pin1 has been reported to promote tumorigenesis through upregulation of numerous cancer-driving pathways. Although Pin1 is a key drug target for treating acute promyelocytic leukemia (APL) caused by a fusion oncogene, much less is known about the role of Pin1 in other heterogeneous leukemia.

**Methods:**

The mRNA and protein levels of Pin1 were detected in samples from de novo leukemia patients and healthy controls using real-time quantitative RT-PCR (qRT-PCR) and western blot. The establishment of the lentiviral stable-expressed short hairpin RNA (shRNA) system and the tetracycline-inducible shRNA system for targeting Pin1 were used to analyze the biological function of Pin1 in AML cells. The expression of cancer-related Pin1 downstream oncoproteins in shPin1 (Pin1 knockdown) and Pin1 inhibitor all-trans retinoic acid (ATRA) treated leukemia cells were examined by western blot, followed by evaluating the effects of genetic and chemical inhibition of Pin1 in leukemia cells on transformed phenotype, including cell proliferation and colony formation ability, using trypan blue, cell counting assay, and colony formation assay in vitro, as well as the tumorigenesis ability using in vivo xenograft mouse models.

**Results:**

First, we found that the expression of Pin1 mRNA and protein was significantly increased in both de novo leukemia clinical samples and multiple leukemia cell lines, compared with healthy controls. Furthermore, genetic or chemical inhibition of Pin1 in human multiple leukemia cell lines potently inhibited multiple Pin1 substrate oncoproteins and effectively suppressed leukemia cell proliferation and colony formation ability in cell culture models in vitro. Moreover, tetracycline-inducible Pin1 knockdown and slow-releasing ATRA potently inhibited tumorigenicity of U937 and HL-60 leukemia cells in xenograft mouse models.

**Conclusions:**

We demonstrate that Pin1 is highly overexpressed in human AML and is a promising therapeutic target to block multiple cancer-driving pathways in AML.

**Electronic supplementary material:**

The online version of this article (10.1186/s13045-018-0611-7) contains supplementary material, which is available to authorized users.

## Background

Acute myeloid leukemia (AML) is the most common form of acute leukemia and arises from a malignant transformation of multipotent hematopoietic stem cells with a remarkable genomic alteration [1]. AML development requires the collaboration of at least two classes of cytogenetic abnormalities [2]. This “two-hit model” [3], presented by Gilliland and Griffin (2002), proposes that class I mutations activate signaling transduction pathways to promote cell proliferation and that class II mutations affect transcription factors to block maturation of hematopoietic cells [4, 5]. The proteins involved in AML processes include both fusion proteins (e.g., RUNX1/ETO, CBFβ/MYH11, and PML/RARα) and mutated proteins (e.g., NPM1, FLT3, and C/EPBα Eisfeld, 2017 #11467, [6–9]), as well as some molecular features that are a function of overexpression (e.g., BAALC, MN1, ERG-1, and AF1q) [10]. Such complexity of molecular and cytogenetic abnormalities poses a major challenge to formulating AML therapy [11, 12]. Chemotherapeutic approaches continue to be the mainstay therapies used for AML treatments [13]. However, long-term survival using these therapies is only obtained in 35 to 40% of younger patients [14, 15], and the long-term survival of elderly AML patients is even lower because only about one third of them are eligible for intensive chemotherapies [16]. Therefore, therapy still needs to come a long way to fully overcome AML. Hence, there is a pressing need to identify potent therapeutic targets to block multiple cancer-driving pathways for the treatment of AML.

Pin1 is a unique peptidyl-prolyl isomerase (PPIase) that catalyzes *cis*/*trans* isomerization of specific pSer/Thr-Pro motifs and central common phosphorylation motifs in cell proliferation and transformation [17]. Hence, altered Pin1 function can play a profound role in pathogenesis of human disease, notably cancer. Pin1 is overexpressed and/or over-activated in many human cancers, thereby disrupting the balance between oncoproteins and tumor suppressors, and by amplifying numerous cancer-driven pathways. Moreover, overexpression and/or over-activation of Pin1 frequently correlate with poor clinical prognosis [17–24].

Besides the well-known role of Pin1 in tumorigenesis, recent studies have revealed that Pin1 dysregulation plays an important role in cancer stem cells (CSCs) in breast cancer and leukemia [25–28]. In primary human breast tumors, the expression of Pin1 is 5 and 30 times higher in non-CSC tumor cells and CSCs, respectively, compared with normal breast epithelial cells [26]. Mechanically, Pin1 promotes breast cancer stem cell (BCSC)-proliferation and tumorigenesis in vitro and in vivo by increasing Rab2A transcription and thereby Erk activation, Zeb1 upregulation, and β-catenin nuclear translocation [25]. Moreover, Pin1 can also stabilize NOTCH1 expression by reducing ubiquitin ligase FBW7 to promote self-renewal and metastasis in breast cancer CSCs [27, 29]. In acute promyelocytic leukemia (APL), genetic or chemical inhibition of Pin1 can induce the degradation of the disease-causing fusion oncogene PML-RARα, which causes APL through blockage of promyelocyte differentiation and promotion of self-renewal capacity [28]. In addition, Pin1 is required not only for the stability of the stem cell reprogramming factors Nanog, Oct4, and MYC but also for inducing and maintaining pluripotency [28, 30–33]. Therefore, Pin1 dysregulation has an important role in the self-renewal and tumorigenic properties of CSCs.

Given that Pin1 can promote tumorigenesis by activating multiple signaling pathways and inducing and sustaining self-renewal capability in a variety of solid tumors, Pin1 may also play a critical role in leukemogenesis. Pin1 is a key drug target for treating APL [28], and controls Notch3 protein expression and regulates T-ALL progression [34]. Silencing Pin1 can delay the progression of lymphoma disease in Eμ-myc transgenic mice; however, much less is known about the role of Pin1 in the development and treatment of other more common and heterogeneous leukemia.

In this manuscript, our clinical data analyses demonstrated higher Pin1 expression in a variety of AML subtypes compared to healthy controls. Genetic or chemical inhibition of Pin1 downregulated multiple cancer-promoting signaling pathways, leading to the inhibition of cell proliferation and colony formation capability in multiple human AML cell lines. Moreover, inducible downregulation or chemical inhibition of Pin1 inhibited the tumorigenesis of AML in vivo. Taken together, these results demonstrate that Pin1 is highly overexpressed in human AML and is a promising therapeutic target to block multiple cancer pathways in AML.

## Methods

### Sample source

Bone marrow samples (*n* = 150) were collected from healthy donors and patients treated in the Hematology Department of the Fujian Medical University Union Hospital from September 2012 to February 2015. Of these, 107 samples were taken from patients with acute leukemia (AL) and 43 were taken from healthy donors. AL patients after the first visit included 61 male and 46 female patients in age of 13~76 years old with median age of 45 years old. According to FBA classification for the diagnosis and classification of AML [35], there were 6 patients with M0, 9 with M1, 18 with M2, 7 with M3, 1 with M4, 41 with M5, 3 with M6, 1 with M7, 21 cases with other types of AL, 43 healthy controls included 20 male, and 23 female donors in age of 20~45 years old with median 32 years old. All of the patients and healthy donors signed informed consent.

### Cell strain and reagents

All cells were cultured in RPMI1640 with 10 or 20% FBS. HL60 (acute myelogenous leukemia cell line), U937 (histiocytic lymphoma cell line), KG-1a (acute myeloid leukemia cell line), NB4 (acute promyelocytic leukemia cell line), Nalm-6 (acute B acute lymphoblastic leukemia cell line), Molt-4 (acute T acute lymphoblastic leukemia cell line), Kasumi-1 (acute myelogenous leukemia cell line), and K562 (chromic granulocytic leukemia cell line) were cultured at the Fujian Institution of Hematology. HL-60, U937, and KG-1a cell lines were cultured at the BIDMC and were gifts from Dr. Daniel G Tenen. Antibodies against various proteins were obtained from the following sources: Pin1 was previously described [36]; β-actin from Sigma; Cyclin D1 (DCS-6) from Biolegend; NF-κB/p65 (D14E12, 8242) from Cell Signaling Technology; β-catenin from BD biosciences; and Rab-2A (15420-1-AP) from Proteintech Group. All-trans retinoic acid (ATRA) were purchased from Sigma, 1,25-dihydroxyvitamin D3 were purchased from Cayman Chemicals, and ATRA-releasing pellets were from Innovative Research of America.

### Quantitative real-time PCR

Total RNA from bone marrow mononuclear cell and cell line was extracted by TRIzol (Invitrogen), and cDNA was transcribed with reverse transcription kit (Thermo Fisher Scientific) in accordance with the manufacturer’s protocols. PCR reactions were performed using quantitative PCR kit (Roche) and ABI7500 fluorogenic quantitative PCR instrument (Applied Biosystem Company). β-actin was used as an internal control of samples. The following primers were used:

- β-actin (F): AGTGTGACGTGGACATCCGCAA.
- β-actin (R): ATCCACATCTGCTGGAAGGTGGAC.
- Pin1 (F): GCTCAGGCCGAGTGTACTACTT.
- Pin1(R): CGAGGCGTCTTCAAATGG.

In line with the operation steps of the quantitative PCR kit (Roche), the reaction system contains SYBR Green Master (ROX) 10 μl, upstream primer (10 pmol/μl) 0.15 μl, downstream primer (10 pmol/μl) 0.15 μl, cDNA 1 μl, and double distilled water 8.7 μl. The reaction was conducted in ABI7500 fluorogenic quantitative PCR instrument (Applied Biosystem Company) under conditions: at 50 °C for 2 min and 95 °C for 10 min, and then at 95 °C for 15 s and 60 °C for 1 min as 1 cycle for 40 times in total. Then, melting curve reaction was conducted under conditions: at 95 °C for 15 s, 60 °C for 1 min, 95 °C for 15 s, 60 °C for 15 s, 1 cycle for once. Three ventral orifices were set in every sample. With β-actin as an internal reference, the relative expression quantity of mRNA was represented with RQ value by 2^−ΔCT^ method: RQ = 2^−ΔCT^. The final results are presented as the fold change of Pin1 expression in a target sample relative to a reference sample, normalized to β-actin.

### Lentiviral transduction and knockdown of Pin1 in AML cells

Recombinant lentiviral particles were produced in 293FT cells by co-transfecting pRevTRE plasmid containing Pin1 shRNA sequence, along with helper plasmids, including pCMV-VSVG and pCMV-dR8.91. The virus-containing medium was harvested at 48 h after transfection and filtered using a 0.45-μm filter. For infection, the collected virus medium was added to the rtTA-expressing cells with polybrene. The cells were selected with G418 and puromycin. Pin1 shRNA were induced by doxycycline, and Pin1 protein level was analyzed by immunoblot. Pin1 shRNA sequence is CCACCGTCACACAGTATTTAT.

### Cell viability assay

Cells were seeded on 96-well plates at a density of 5000 cells per well. Cells were stained with Trypan blue and counted every day. In two additional groups, cells were treated with DMSO or ATRA. After 72 h, cell viability was measured by CCK-8 assay (DOJINDO) and the number of cells was determined by CellTiter-Glo® 2.0 Assay (Promega, Madison, WI) according to the manufacturer’s instructions.

### Colony formation assay

Cells were seeded on 24-well plate at a density of 500 cells (KG-1a is 1000 cells). Methylcellulose (Sigma) in final concentration of 0.8% was added and mixed. After 7 days (KG-1a is 14 days), cells were stained with 0.5 ml 1 mg/ml p-Iodonitrotetrazolium Violet. The number of colonies and the total area were calculated by ImageJ analysis.

### PPIase assay

The PPIase activity on GST-Pin1 in response to ATRA and 1,25-(OH)_2_ vitamin D3 were determined using the chymotrypsin-coupled PPIase activity assay with the substrate Suc-Ala-pSer-ProPhe-pNA (50 nM) in buffer containing 35 mM HEPES (pH 7.8) and 0.1 mg/ml BSA at 10 °C as described, with the exception that the compounds were preincubated with enzymes for 12 h at 4 °C [37].

### FACS analysis

To assess cell surface expression of CD11b, cells were washed with PBS, harvested by non-enzymatic cell dissociation solution, and resuspended in blocking solution (Ca2+, Mg2+-free PBS containing 2% FCS). Cells were then incubated with CD11b-FITC (Biolegend) for 15 min at 4 °C in the dark. Cells were washed and analyzed by using a Beckman Coulter’s Gallios flow cytometry.

### Western blot

Cells from each group were collected, and cell lysis buffer (Roche Company) was added to extract total protein. The protein concentration was detected by the Bradford method. The loading sample of equivalent protein was transferred to a nitrocellulose membrane (Bio-Rad, cat. No. 162-0115) through SDS-PAGE electrophoresis and immunoblotting.

### Animal studies

For xenograft experiments, 5 × 10^5^ HL-60 and U937 cells stably expressing Tet-On shPin1 were injected subcutaneously into flank of 7-week-old BALB/c nude mice (Taconic Laboratories) and fed with normal or doxycycline-containing diet. For ATRA treatment, 5 × 10^5^ U937 cells were injected subcutaneously into flank of 7-week-old BALB/c nude mice. After 5 days when tumor growth was just about notable by sight, the mice were randomly divided into placebo group or ATRA slow-releasing pellet group. Tumor growth was monitored twice a week until sacrifice criteria were met in the first mice. Tumor sizes were recorded twice a week by a caliper and tumor volumes were calculated using formula *L* × *W*^2^ × 0.52, where *L* and *W* represent length and width, respectively. Tumors were cut into small pieces and quickly stored in liquid nitrogen, partly for protein extraction. All BALB/c nude mice were housed in laminar flow cabinets with free access to food and water. Animal work was carried out in compliance with the ethical regulations approved by the Animal Care Committee, Beth Israel Deaconess Medical Center, Boston, USA.

### Statistical analysis

Experiments were routinely repeated at least three times, and the repeat number was increased according to effect size or sample variation. Statistical analyses were performed using SPSS 23.0 and GraphPad Prism 5 software package. All data are presented as the means ± SD/SEM, followed by determining significant differences using the two-tailed student’s *t* test or analysis of variance (ANOVA) test where *p < 0.05, **p < 0.01, and ***p < 0.001.

## Results

### Pin1 overexpression in leukemia patients

**Fig. 1:**
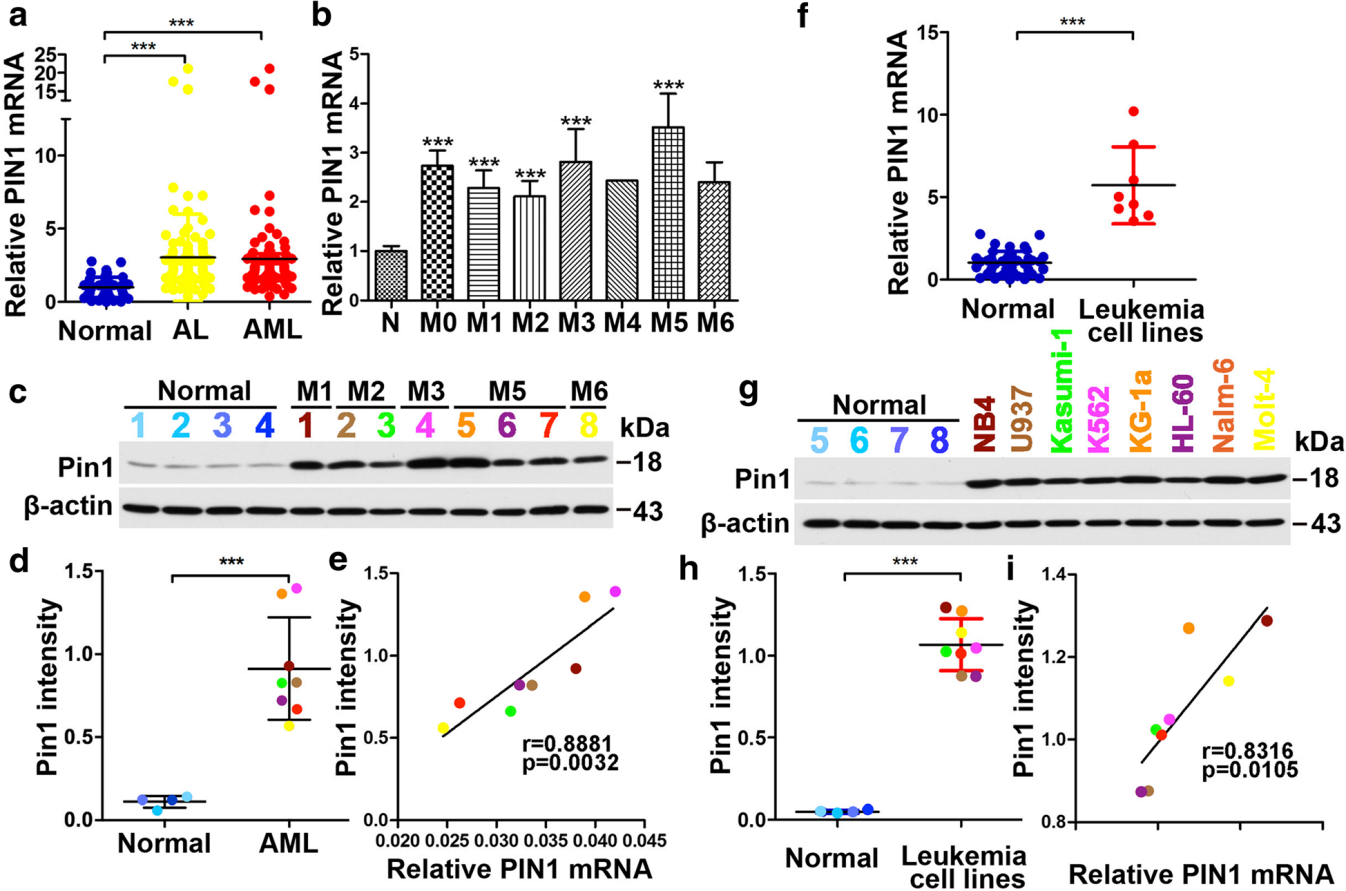
Expression of PIN1 mRNA and protein in leukemia patients and cell lines. **a** The fold changes of PIN1 mRNA expression in bone marrow cells from normal controls, untreated acute leukemia patients (AL) and AML patients. **b** The fold changes of PIN1 mRNA expression in bone marrow cells from normal controls and FAB subtypes of untreated AML patients. **c**, **d** Pin1 protein levels in bone marrow cells from normal and AML patients were analyzed by immunoblotting (**c**). The quantitative results of Pin1 expression was analyzed from 1c (**d**). The individual patients used for immunoblot analysis were indicated by different colors. **e** The correlation between PIN1 mRNA and Pin1 protein levels in AML patients. Different colors were used to indicate different patients in 1c and 1d. **f** The fold changes of PIN1 mRNA expression in normal control bone marrow cells and several leukemia cell lines, including AML cells (Kasumi-1, U937, K562, NB4 and KG-1a) and ALL cells (Nalm-6 and Molt-4). **g**, **h** The protein levels (**g**) and quantitative results (**h**) of Pin1 in normal control bone marrow cells and leukemia cell lines. Different cell lines were indicated by corresponding colors. **i** The correlation between PIN1 mRNA and Pin1 protein levels in leukemia cell lines was analyzed based on 1f and 1 h. Individual cell lines were indicated by corresponding colors. Statistically significant differences using Student’s *t* test are indicated by *p* values. (*p < 0.05, **p < 0.01, ***p < 0.001)

To investigate the potential clinical significance of Pin1 in leukemia, we examined the relative expression of PIN1 mRNA in bone marrow mononuclear cells from 107 newly diagnosed leukemia patients and 43 healthy bone marrow donors. We found that PIN1 mRNA expression was significantly higher in bone marrow cells of acute leukemia (AL) patients compared to those of healthy controls (Fig. 1a, lane 2 versus lane 1). Among these samples, 86 samples were diagnosed with AML patients, which also showed significantly higher PIN1 mRNA expression, as compared with healthy controls (Fig. 1a, lane 3 versus lane 1). Further analysis of the relationship between the expression of PIN1 mRNA and FAB (French–American–British classification system) subtypes of AML showed that PIN1 mRNA was upregulated in most subtypes of AML. In the case of M4 and M6, low sample sizes precluded sufficient power for statistical analysis (Fig. 1b). Consistent with the results of PIN1 mRNA, Pin1 protein levels were higher in AML patient samples compared with healthy controls (Fig. 1c, d; Additional file 1: Figure S1a). A positive correlation was found between PIN1 mRNA level and Pin1 protein level (Fig. 1e). In addition, we used different colors for individual patients in western blot analysis. These results together demonstrate that Pin1 is highly overexpressed in AML patients.

We also examined Pin1 expression in human leukemia cell lines, including six AML cell lines (Kasumi-1, U937, K562, NB4, HL-60, and KG-1a) and two ALL cell lines (Nalm-6 and Molt-4). Both PIN1 mRNA (Fig. 1f) and Pin1 protein levels (Fig. 1g, h; Additional file 1: Figure S1b) were found to be significantly higher in human leukemia cell lines as compared with bone marrow cells of healthy control (Fig. 1f–h). A positive correlation of PIN1 mRNA and Pin1 protein levels was also found in leukemia cell lines (Fig. 1i). These results further confirm that Pin1 is overexpressed in leukemia cells including AML cells.

### Constitutive Pin1 knockdown inhibits cell proliferation and clonogenicity of human AML cells in vitro

**Fig. 2:**
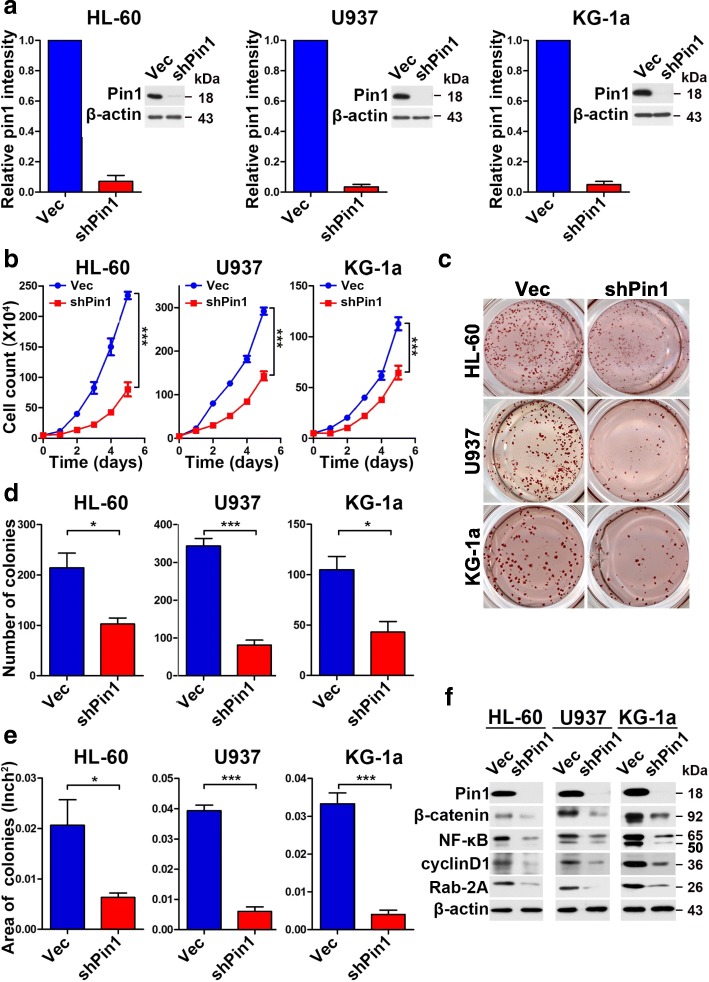
Constitutive Pin1 downregulation suppresses oncogenic biological functions and signaling in vitro. **a** To establish stable-shPin1 cell lines, HL-60 or U937 or KG-1a cells were infected with lentivirus expressing scramble (Vec) or Pin1 shRNA (shPin1) with Puro^r^. After puromycin selection for 1 week, Pin1 levels were validated by immunoblotting analysis. **b** Pin1 downregulation inhibits cell proliferation in indicated AML cell lines. Cell growth was monitored for 1 week by cell counting. *p* values were derived from the cell numbers for each group at the end point. **c–e** Cells were cultured in normal medium supplemented with methylcellulose for 1 or 2 weeks. When colonies became visible, cells were stained with *p*-iodonitrotetrazolium violet for counting. The number (**d**) and area (**e**) of colonies was measured and counted using ImageJ. Results present the mean ± SD of three independent experiments. **f** Cell lysates were subjected to western blot analysis with antibodies against the downstream oncogenic proteins of Pin1. Statistically significant differences using Student’s *t* test are indicated by *p* values. (*p < 0.05, **p < 0.01, ***p < 0.001)

Given the overexpression of Pin1 in AML, including biopsied bone morrow leukemia cells and established human AML cell lines, the question remains as to whether Pin1 plays any role in leukemogenesis. Based on the “two-hit model,” the ability of a molecule to promote cell proliferation and colony formation is required in order to initiate leukemogenesis [38]. We investigated the effects of Pin1 on leukemogensis-related traits. To this end, multiple AML cell lines, including HL-60, U937 and KG-1a, were infected with lentiviruses carrying validated Pin1-specific shRNA [26] or control shRNA with Puro^r^. Using puromycin selection, stable Pin1-knockdown (Pin1 KD) cell lines were established. Immunoblot analysis confirmed a dramatic decrease in endogenous Pin1 in cells carrying Pin1-shRNA compared with cells carrying the control shRNA (Fig. 2a).

The rates of cell growth of different pairs of AML Pin1-shRNA and control-shRNA cells were monitored by counting cell number to demonstrate the effects of Pin1 downregulation on cell proliferation. In all three AML cell lines examined, Pin1 KD resulted in a significant reduction in cell proliferation (Fig. 2b).

To test if Pin1 KD would impair the leukemogenic potential of AML cells to grow as clonogenic colonies, HL-60, U937, and KG-1a expressing Pin1 shRNA or control shRNA were plated at a low density and observed for colony formation. A significant decrease both in the number (Fig. 2c, d) and size (Fig. 2c, e) of colonies was observed in the Pin1 KD group compared with controls. Thus, our data demonstrates that Pin1 plays a positive role in leukemogenesis-relevant events in vitro. As a major regulator of oncoproteins, Pin1 amplifies oncogenic pathways by activating more than 43 oncogenic molecules and suppressing at least 20 tumor-suppressing molecules, including many of which have well-established roles in CSCs [17, 21, 39]. To further characterize the underlying mechanisms of Pin1-mediated leukemogenesis, we analyzed the expression of several leukemogeneis-related signaling molecules that have been reported to be Pin1 substrates. Wnt/β-catenin and NF-κB pathways are well-known signaling pathways in leukemogenesis through regulating cell proliferation, differentiation, or apoptosis and have been shown to be Pin1 substrates in other cancer cells [26, 40–46]. AML Pin1 KD cells also downregulated the expression of Pin1 oncoprotein substrates, including β-catenin and NF-κB (Fig. 2f), as with solid tumors [26, 40–46]. Besides these key regulators in leukemogenesis, Pin1 also transcriptionally regulated some stem cells-related molecules, including Rab2A [47] and CyclinD1 [48, 49]. In AML Pin1 KD cells, these molecules were downregulated as well (Fig. 2f) (Additional file 2: Figure S2). Therefore, these biochemical analyses further support the role for Pin1 in the regulation of cell proliferation and clonogenicity through deregulation of multiple oncogenic pathways.

### Doxycycline-inducible Pin1 knockdown inhibits clonogenicity of human AML cells in vitro

**Fig. 3:**
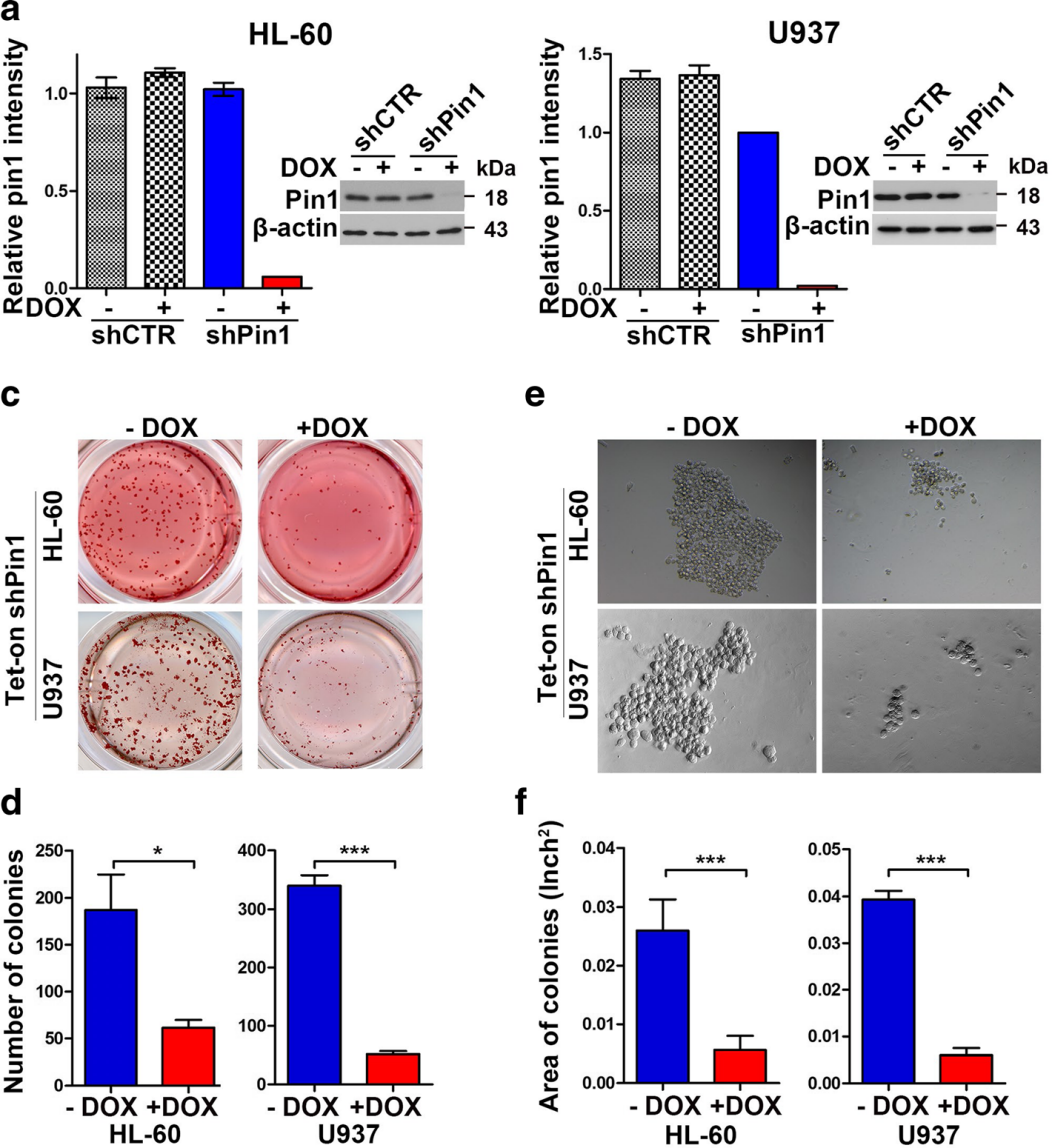
Doxycycline-induced Pin1 downregulation in HL-60- and U937-Tet ON cells suppress clonogenicity in vitro. **a**, **b** Using Tet-On system, we generated inducible-shPin1 HL-60 (**a**) or U937 (**b**) cells. The effects of Pin1 downregulation were assayed by immunoblotting after Dox treatment (1 mg/ml) for 3 days. **c–f** Cells were cultured in normal medium supplemented with methylcellulose for 1 or 2 weeks. When colonies became visible, the morphology of cells were taken by transmission electron microscopy (**c**), followed by staining with *p*-iodonitrotetrazolium violet for counting (**d**). The number (**e**) and area (**f**) of colonies was measured and counted using ImageJ. Results present the mean ± SD of three independent experiments. Statistically significant differences using Student’s *t* test are indicated by *p* values. (*p < 0.05, **p < 0.01, ***p < 0.001)

To further confirm and evaluate the effects of Pin1 inhibition on the tumorigenesis of AML, we generated stable tetracycline-inducible Pin1 knockdown HL-60 and U937 cells using a Tet-On system [28, 50]. We infected HL-60- and U937-rtTA cells with lentivirus containing either Pin1 shRNA or control shRNA with Puro^r^. Stable tetracycline-inducible Pin1 KD and control HL-60- and U937-Tet ON cells were established using puromycin selection. In the presence of doxycycline, Pin1 protein levels were dramatically reduced in both cell lines (Fig. 3a, b). Furthermore, the clonogenic assay showed a significant decrease in the number (Fig. 3c, d) and size of colonies (Fig. 3e, f) after doxycycline treatment as compared with the vehicle control. These results show that our Tet-On Pin1 KD system is highly inducible and could be used to confirm the role of Pin1 in leukemogenesis, as shown in constitutive Pin1 KD cells (Fig. 2).

### Doxycycline-inducible Pin1 knockdown inhibits tumorigenesis of human AML cells in vivo.

**Fig. 4:**
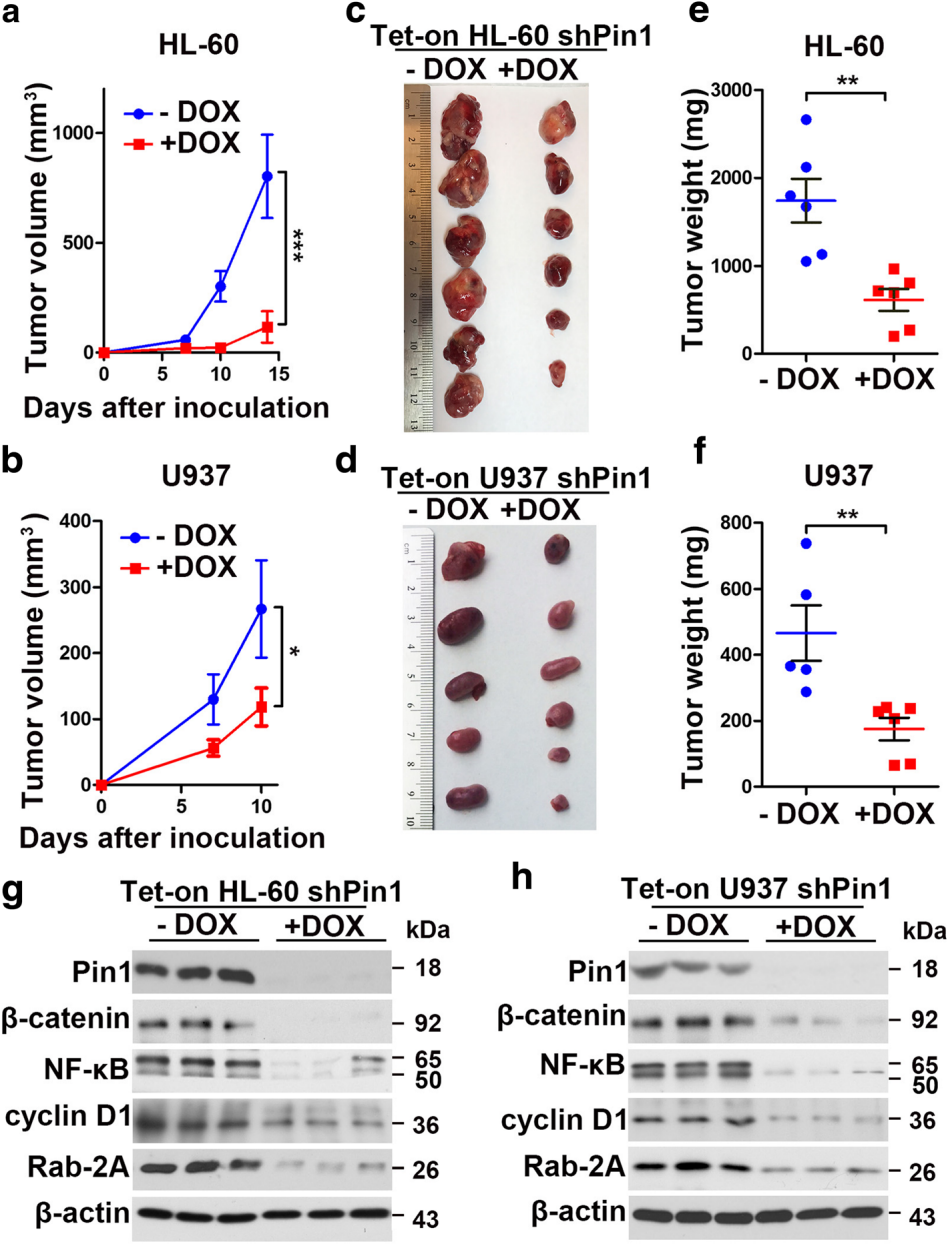
Pin1 knockdown suppresses tumor growth of human leukemia cells in vivo. After treating HL-60 and U937 Tet-On cells with or without Dox (1 mg/ml) for 3 days in vitro, cells were subcutaneously implanted into the flanks of nude mice fed with a normal or a doxycycline-containing diet,respectively. **a**, **b** Tumor volumes were measured and the tumor growth curves were plotted over time. Error bars represent standard deviations. **c**, **d** Photographic illustration of tumors harvested from nude mice at the end point. Each scale of the ruler represents 1 mm. **e**, **f** Weights of tumors harvested from nude mice at the end point. Error bar represents SEM. **g**, **h** Total proteins from xenograft tumor samples were subjected to western blot analysis of the indicated proteins. Statistically significant differences using Student’s *t* test are indicated by *p* values. (*p < 0.05, **p < 0.01, ***p < 0.001)

To examine whether inducible Pin1 inhibition would affect tumorigenesis of human AML cells in vivo, we subcutaneously implanted HL-60- and U937-Tet ON cells into the flanks of nude mice, then fed the mice with a normal or a doxycycline-containing diet, respectively. Tumor growth was monitored twice a week until sacrifice criteria were met in the first mice. Our results showed that doxycycline-induced Pin1 KD significantly suppressed tumor growth in both HL-60 (Fig. 4a) and U937 Pin1 KD cells (Fig. 4b) with reductions of both tumor weight and tumor volume exceeding 50% (Fig. 4c–f). Thus, inducible Pin1 downregulation suppressed tumor growth in AML xenograft model in vivo. To assess inactivation of Pin1-regulated cancer pathways in primary xenograft tumors in vivo, we extracted total proteins from xenograft tumor samples and then subjected to perform western blot analyses on Pin1 and its downstream oncoproteins, including β-catenin, NF-κB, cyclinD1 and Rab-2A, whose protein levels have been shown to be upregulated by Pin1 [26, 40–46, 51]. The protein levels of these oncoproteins were decreased with Pin1 downregulation (Fig. 4g, h), implying that Pin1 KD suppresses tumorigenesis through downregulation of oncogenic signaling pathways.

### The Pin1 inhibitor ATRA downregulates Pin1 oncoprotein substrates and inhibits cell growth and clonogenicity of human AML cells in vitro

**Fig. 5:**
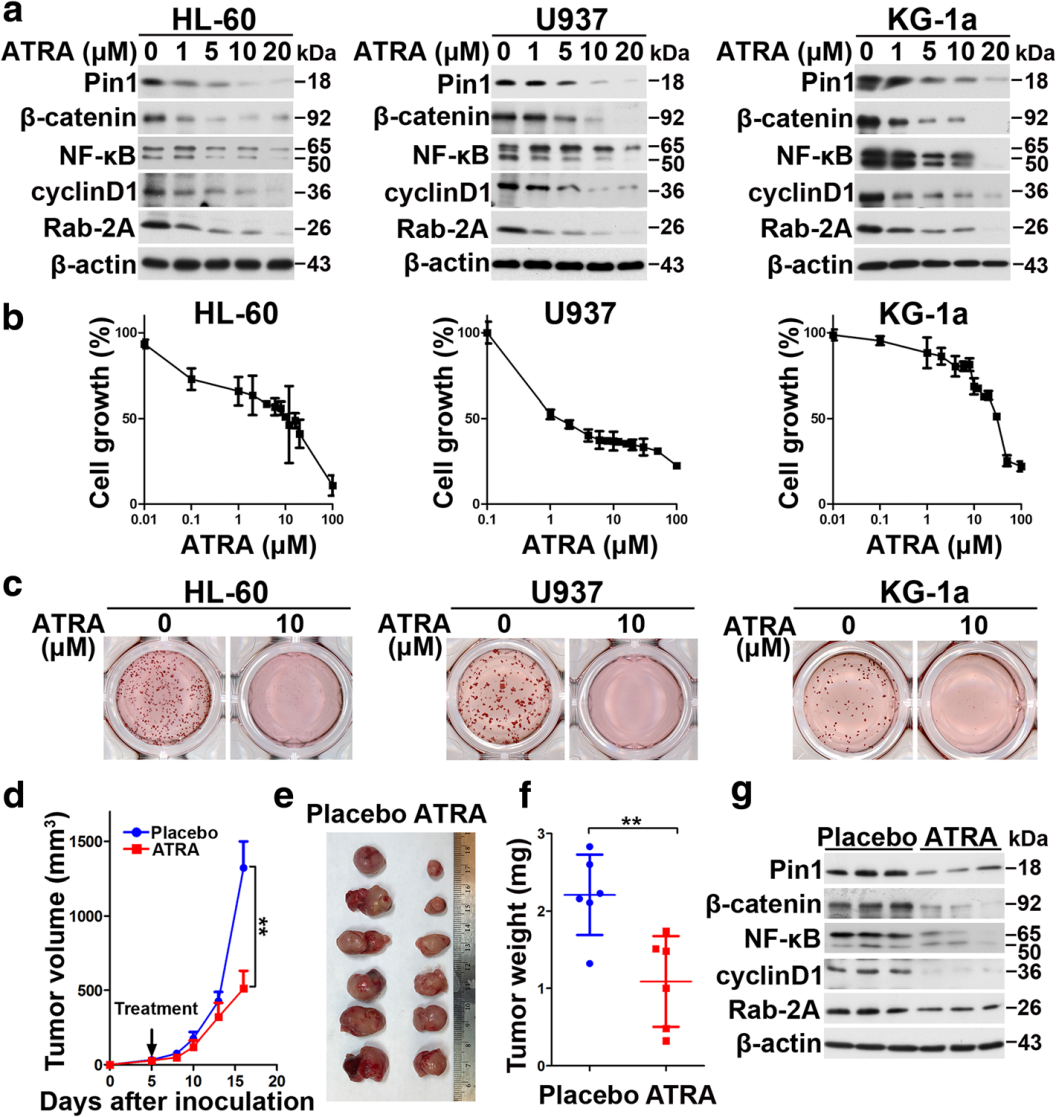
ATRA inhibits the tumorigenesis of human leukemia cells and blocks multiple cancer-driving pathways in vitro and in vivo. **a** After 72 h incubation of different concentrations of ATRA, cell lysates were subjected to western blot analysis of the indicated proteins. **b** ATRA inhibits cell proliferation in indicated AML cell lines. After 3 days treatment of different concentrations of ATRA, cell growth was measured by CCK-8 analyses. **c** ATRA reduces colony formation in indicated AML cell lines. Cells were cultured in normal medium containing methylcellulose with or without ATRA (10 μM) for 1 or 2 weeks, followed by staining with *p*-iodonitrotetrazolium violet. **d–f** U937 cells were injected subcutaneously into flank of 7-week-old BALB/c nude mice, and the mice were randomly divided into placebo group and ATRA slow-releasing pellet group. Tumor volumes were measured and the tumor growth curves were plotted over time (**d**). Error bars represent standard deviations. Photographic illustration of tumors harvested from nude mice at the end point (**e**). Each scale of the ruler represents 1 mm. Weights of tumors harvested from nude mice at the end point (**f**). Error bar represents SEM. **g** Total proteins from xenograft tumor samples were subjected to western blot analysis of indicated Pin1 downstream oncoproteins. Statistically significant differences using Student’s *t* test are indicated by *p* values. (*p < 0.05, **p < 0.01, ***p < 0.001)

Given the oncogenic role of Pin1 in AML, we wondered whether targeting Pin1 with chemical inhibitors would show any therapeutic benefit in the treatment of AML [28]. ATRA has been reported to inhibit and degrade active Pin1, resulting in the blockade of multiple Pin1-regulated oncogenic pathways in APL, breast cancer, and liver cancer [28, 30, 52]. However, the effect of ATRA on AML cells is unknown. Therefore, we treated multiple AML cells, HL-60, U937, and KG-1a, with different concentrations of ATRA. ATRA can degrade Pin1 and its downstream oncoproteins in a dose-dependent manner (Fig. 5a). Notably, in both cells, among these oncoproteins, while NF-κB was slightly upregulated in low concentrations of ATRA likely due to ATRA-induced differentiation, they were downregulated once ATRA concentration was high enough to induce Pin1 degradation. This further supported that ATRA could downregulate multiple oncoproteins through degrading Pin1 in AML.

Since ATRA induces differentiation in various leukemia cell lines, it is unclear whether differentiation would affect Pin1 levels. To address this question, we treated cells with other differentiation-inducing reagent − 1-α, 25-(OH)_2_ vitamin D_3_ (1,25-D3), the active form of vitamin D_3_, which has been shown to induce differentiation in various AML cell lines [53–56]. After 72 h of incubation of 1, 25-D3 or ATRA, differentiation was evaluated by flow cytometry using a general myeloid marker, CD11b. As shown in Figure S3a (Additional file 3; Figure S3a), the percentages of CD11b-positive cells were increased in a dose-dependent manner in 1,25-D3-treated HL60 and U937, but not in KG-1a, likely due to low basal level of PKCβ [57]. The differentiation state of 1,25-D3 was similar to that of ATRA in HL-60 and U937 (Additional file 3: Figure S3b). Using these models, we further analyzed Pin1 stability and activity. The results showed that 1,25-D3 neither reduced Pin1 levels in cells (Additional file 3: Figure S3c) nor inhibited Pin1 PPIase activity in vitro (Additional file 3: Figure S3d). We also checked the stability of Pin1 downstream targets in U937 (Additional file 3: Figure S3e). As shown in Additional file 3: Figure S3e, differentiation-unrelated Rab2A did not show obvious changes. NF-κB and β-catenin showed a slightly upregulated in high doses, and cyclinD1 was slightly downregulated at high doses, which could likely be due to proliferation inhibition. These results show that differentiation-inducing drug per se could not downregulate Pin1 stability and activity, confirming that ATRA-induced Pin1 degradation and downregulation of Pin1 substrates is differentiation-independent. Furthermore, ATRA treatment also significantly inhibited cell proliferation and colony formation of all three leukemia cells (Fig. 5b, c). To explore if ATRA could be effective for those normal cells that do not overexpress Pin1, we treated two immortalized normal blood cells (N1 and N5 cells) with ATRA. First, in both normal cells, Pin1 protein levels were extremely low (Additional file 4: Figure S4a), as we have shown in normal human bone marrow cells (Fig. 1c, g). Importantly, these two normal blood cell lines were completely resistant to ATRA (Additional file 4: Figure S4b). These results are consistent with our previous findings that ATRA selectively targets active Pin1 in breast cancer cells, but not normal breast cells [58]. Thus, besides genetic inhibition, chemical inhibition of Pin1 by ATRA can also inhibit AML leukemogenesis through downregulation of multiple oncogenic pathways.

### Slow-releasing Pin1 inhibitor ATRA exerts potent anticancer activity against AML in vivo

Given the effects of ATRA on cell growth, leukemogenesis, Pin1 protein levels, and the downstream in AML in vitro, a critical question is whether ATRA suppresses AML tumor growth in vivo. We thus examined ATRA in mouse models xenografted with U937 cells. ATRA is extremely light-sensitive and can be metabolized quickly in the liver, with a 45-min half-life in humans. To improve the activity of drug and maintain a constant drug level in the blood, we implanted slow-releasing ATRA pellets, as described in previous studies [28, 52]. As expected, ATRA blocked the tumor growth (*P* = 0.0037) (Fig. 5d) and tumor size and weight (*P* = 0.0057) (Fig. 5e, f). Furthermore, the protein level of Pin1 and Pin1’s oncogenic downstream (Fig. 5g) was downregulated in xenograft tumors from nude mice treated with slow-releasing ATRA. These results demonstrate that ATRA has potent anti-leukemia activity through targeting Pin1 and multi-cancer driving pathway.

## Discussion

Since the heterogeneity of AML is a challenge to clinical therapy, combination therapy is increasingly being explored for alternative means of overcoming biological heterogeneity in AML. Combining one “targeted” agent with other “targeted” agents or with conventional chemotherapy may enhance treatment efficacy. Our studies suggest that Pin1 could be a potent therapeutic target in AML. Not only is Pin1 highly overexpressed in human primary AML cells and established AML cell lines, but the chemical and genetic inhibition of Pin1 potently inhibits leukemogenesis in vitro and tumorigenesis in vivo. Thus, combining Pin1-targeted agents with other chemotherapy agents may provide a more efficient treatment to overcome the heterogeneity of AML.

Elevated expression of Pin1 was detected in acute leukemia patients in our study. The underlying mechanism of increased Pin1 expression likely arises from C/EBPα-p30 which can increase Pin1 mRNA and protein levels through E2F1, resulting in reduced C/EBPα function, blocked cell differentiation and eventual AML [59]. From our own clinical data, we were unable to confirm this correlation due to insufficient numbers of C/EBPα-p30 positive samples. Despite the lack of sufficient C/EBPα-p30 samples, significantly higher levels of Pin1 expression were still detected in AML compared with healthy controls, which implies that C/EBPα-p30 is not the only mechanism of Pin1 regulation.

Several studies have shown that Pin1 can activate multiple oncogenic signaling pathways in solid tumors [17]. We now show that Pin1 can promote tumorigenesis in AML via the activation of multiple oncogenes, including β-catenin and NF-κB molecules, which belong to the Wnt/β-catenin and NF-κB pathways, respectively. All of these pathways are deregulated in AML leukemogenesis [60–62]. In particular, The Wnt signaling pathway is essential for the maintenance of hematopoietic stem cells, and the fine-tuning regulation of β-catenin expression levels is important for hematopoiesis progression [63, 64]. The NF-κB signaling pathway also plays an important role in the development of AML [65]. Around 40% of AML patients have high expression of constitutive NF-κB, the aberrant activity of which stimulates leukemia cell proliferation and prevents leukemia cell apoptosis, leading to leukemogenesis [66]. Given the fundamental role of these signaling pathways in AML leukemogenesis, more and more therapeutic agents targeting these molecules are being developed [67–70]. Combining these targeted agents with Pin1 inhibitors, for example ATRA, may result in even more efficacious treatment of AML patients.

In addition to its role in cell proliferation, the regulation of leukemic colony formation is another biological phenomenon affected by Pin1 in leukemogenesis. Silencing Pin1 by either a stable-shRNA system or an inducible-shRNA system reduced colony numbers of AML cells in vitro. Furthermore, silencing Pin1 downregulated β-catenin in AML cells both in vitro and in vivo. Wnt/β-catenin signaling is known to be involved in the establishment of leukemia stem cells [71]. Leukemia stem cells are hypothesized to be responsible for driving leukemia development and disease relapse [72]. The expression of β-catenin is also correlated with the clonogenic proliferation of AML cells and poor prognosis [73, 74]. This implies that Pin1 can maintain the leukemia stem cell population and therefore represent an ideal target for the effective treatment of AML.

In our studies, Pin1 degradation is not related to the effect of leukemia cell differentiation. When we treated AML cells with other differentiation-inducing reagent - vitamin D3, Pin1 protein levels remain unchanged. These results further support that ATRA-induced Pin1 degradation is through direct-binding [58]. On the other hand, unlike the beneficial effects of differentiation induction in APL, it is difficult to predict the effects of differentiation induction in individual AML patients [75–77]. Therefore, to identify other agents targeting key molecules in AML, such as Pin1, in combination with differentiation induction therapy would be another strategy to cure AML.

ATRA has been identified as a Pin1 inhibitor, but it had limited success in treatment of non-APL-AML and the results of clinical trial have been overall disappointing [78]. ATRA binds and induces degradation of Pin1 and its substrate PML-RARα and thereby exerts anticancer activity against APL in cell and animal models and human patients [28]. This anticancer activity of ATRA has been confirmed and expanded to breast and liver cancer, but only when slow-releasing ATRA formulations that maintain a constant drug concentration is used [52]. Here, we have shown an anti-leukemia activity against AML for ATRA in vitro and in mice when slow-releasing ATRA pellets are used. In contrast to free ATRA, these pellets are able to maintain ATRA serum concentrations in mice constant at the concentrations for Pin1 binding and inhibition as described previously [28]. The potent activity of ATRA in inducing Pin1 degradation and inhibiting its oncogenic function in vitro and in vivo has been confirmed using another independent formulation of controlled release ATRA [79]. Similarly, while regular ATRA needs to combine with others to treat APL, 13-year follow-up data show that liposomal ATRA with a longer half-life has significant efficacy in APL patients as a single-agent front-line therapy [80]. These might be related to the fact that ATRA, a vitamin A derivative, is metabolized rapidly in the liver with a very short half-life of 45 min [81–84], possibly because it is an endogenous Pin1 inhibitor [28]. This idea is also consistent with the clinical findings that regular ATRA has some detectable but not striking results against AML [78] or solid tumors, but second and third generations of much more stable and potent retinoid derivatives show little efficacy [85], likely because they potently target RARs or RXRs, but no longer bind to Pin1 [28, 86]. Thus, there is an urgent need to develop a longer half-life ATRA formulation or Pin1-targeted ATRA derivatives or more specific Pin1 inhibitors for treating non-APL leukemia and other cancers.

## Conclusion

Taken together, we have shown that Pin1 is overexpressed in most human AML patient tissues and cell lines, and that genetic and chemical inhibition of Pin1 inhibits cell proliferation and colony formation and leukemogenesis in AML in vitro and in mice through blocking multiple oncogenic signaling pathways. Thus, our findings provide evidence that Pin1 is a potential therapeutic target for AML and suggest that the development of longer half-life ATRA or more potent and specific Pin1 inhibitors could prove effective in the treatment of AML.

## Additional files

Additional file 1: **Figure S1.** The original whole blots of Pin1 expression in AML patients and leukemia cell lines. **a** The stronger signals of Pin1 protein levels of healthy controls and AML patients were detected with a 5 min exposure of blot than with a 30 s exposure of blot. **b** The stronger signals of Pin1 protein levels of healthy controls and leukemia cell lines were detected with a 5 min exposure of blot than with a 30 s exposure of blot. (PDF 1205 kb)

Additional file 2: **Figure S2.** The relative intensities of Pin1 downstream oncoproteins in Vec and shPin1. The intensities of each protein signal were determined by ImageJ. The semi-quantitative results were averaged from three independent experiments. (PDF 312 kb)

Additional file 3: **Figure S3.** 1,25-(OH)_2_VitaminD_3_, does not affect Pin1 stability and function. **a, b** The expression of CD11b were assayed by FACS in HL-60, U937 and KG-1a at 72 h after treatment (a). 1, 25-D3 induces HL-60 and U937 differentiation, but not KG-1a. The differentiation state of each cell was assayed by the percentages of CD11b positive cells in indicated cell lines (b). **c** Pin1 protein levels were not changed after 72 h incubation of 1, 25-D3 in HL-60 and U937. **d** 1,25-D3 does not inhibit PPIase activity of Pin1. Pin1 was incubated with different concentrations of 1, 25-D3, followed by chymotrypsin-coupled PPIase assay. **e** Pin1 downstream oncoproteins were assayed after 72 h incubation of 1,25-D3 in U937. (PDF 2712 kb)

Additional file 4: **Figure S4.** Immortalized normal blood cells were resistant to ATRA. **a** Pin1 protein levels in two immortalized normal blood cells (N1 and N5 cells) were assayed by immunoblotting and compared with AML cell lines (HL-60, U937 and KG-1a). N was indicated normal blood cells. **b** After 3 days treatment of different concentrations of ATRA, cell growth rates were determined by CellTiter-Glo® 2.0 Assay. N1 and N5 cells were completely resistant to ATRA, compared with leukemia cell lines. (PDF 222 kb)

## Abbreviations

1,25-D3: 1-α, 25-Dihydroxyvitamin D_3_
AL: Acute leukemia
ALL: Aacute lymphoblastic leukemia
AML: Acute myeloid leukemia
APL: Aacute promyelocytic leukemia
ATCC: American Type Culture Collection
ATRA: All-trans retinoic acid
BCSC: Breast cancer stem cell
CCK-8: Cell Counting Kit-8
CSCs: Cancer stem cells
Dox: Doxycycline
FAB: French–American–British classification systems
Pin1 KD: Pin1-knockdown
Pin1: Peptidyl-prolyl *cis*-*trans* isomerase NIMA-interacting 1
PPIase: Peptidyl-prolyl isomerase
RT-qPCR: Real-time quantitative PCR
rtTA: Reverse tetracycline-controlled transactivator
SDS–PAGE: Sodium dodecyl sulfate polyacrylamide gel electrophoresis
shPin1: Pin1 shRNA
shRNA: Short hairpin RNA
Tet: Tetracycline
Vec: Vector
WB: Western blot
