# Correction to: Pin1 inhibition exerts potent activity against acute myeloid leukemia through blocking multiple cancer-driving pathways

**DOI:** 10.1186/s13045-018-0634-0

**Published:** 2018-07-11

**Authors:** Xiaolan Lian, Yu-Min Lin, Shingo Kozono, Megan K. Herbert, Xin Li, Xiaohong Yuan, Jiangrui Guo, Yafei Guo, Min Tang, Jia Lin, Yiping Huang, Bixin Wang, Chenxi Qiu, Cheng-Yu Tsai, Jane Xie, Ziang Jeff Gao, Yong Wu, Hekun Liu, Xiao Zhen Zhou, Kun Ping Lu, Yuanzhong Chen

**Affiliations:** 1 Fujian Institute of Hematology, Fujian Provincial Key Laboratory on Hematology, Fujian Medical University Union Hospital, Fuzhou, China; 2 Division of Translational Therapeutics, Department of Medicine and Cancer Research Institute, Beth Israel Deaconess Medical Center, Harvard Medical School, Boston, USA; 3 Fujian Key Laboratory for Translational Research in Cancer and Neurodegenerative Diseases, Institute for Translational Medicine, Fujian Medical University, Fuzhou, China

## Correction

The original article [1] contained minor typesetting errors affecting the following authors: Ziang Jeff Gao, Xiao Zhen Zhou, and Kun Ping Lu. The article has now been corrected to display the authors’ names correctly.
