# Supplementary figures and images for: Pin1 inhibition exerts potent activity against acute myeloid leukemia through blocking multiple cancer-driving pathways
Source: J Hematol Oncol. 2018 May 30;11:73. doi: 10.1186/s13045-018-0611-7 (PMC5977460; doi:10.1186/s13045-018-0611-7)

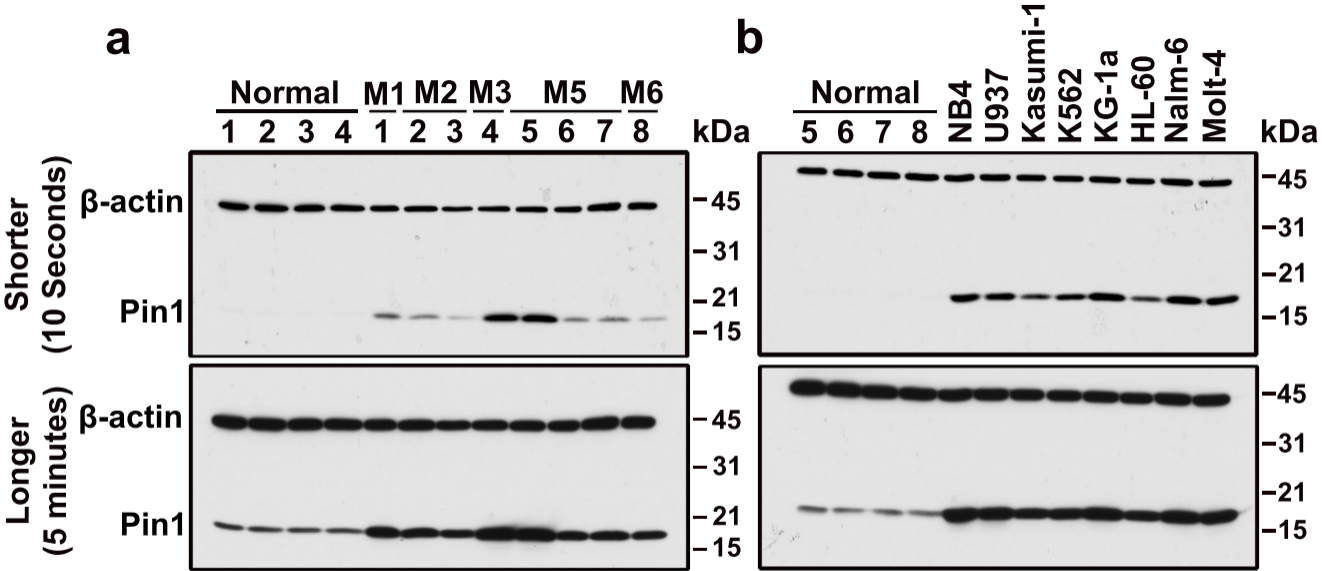

**Supplementary Figure 1**

Supplement: Supplementary file 1 — Figure S1. The original whole blots of Pin1 expression in AML patients and leukemia cell lines. a The stronger signals of Pin1 protein levels of healthy controls and AML patients were detected with a 5 min exposure of blot than with a 30 s exposure of blot. b The stronger signals of Pin1 protein levels of healthy controls and leukemia cell lines were detected with a 5 min exposure of blot than with a 30 s exposure of blot. (PDF 1205 kb) [file 13045_2018_611_MOESM1_ESM.pdf]

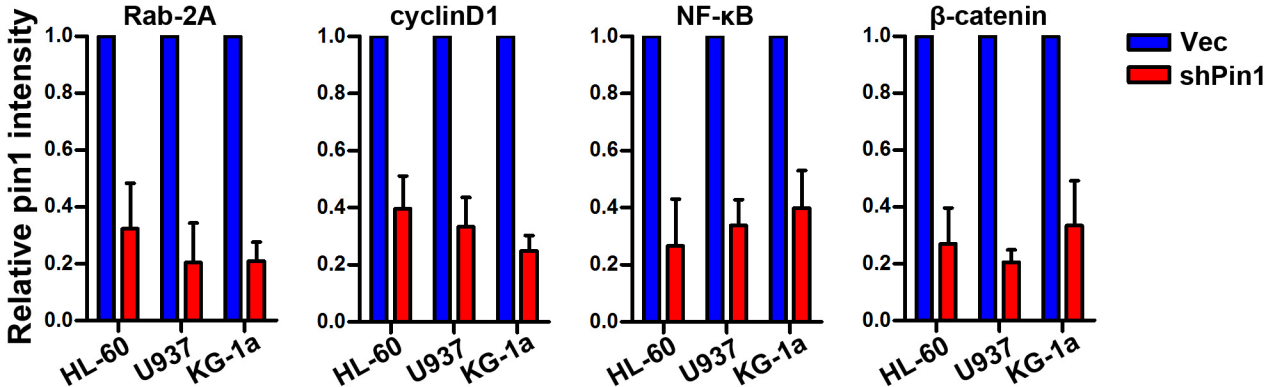

**Supplementary Figure 2**

Supplement: Supplementary file 2 — Figure S2. The relative intensities of Pin1 downstream oncoproteins in Vec and shPin1. The intensities of each protein signal were determined by ImageJ. The semi-quantitative results were averaged from three independent experiments. (PDF 312 kb) [file 13045_2018_611_MOESM2_ESM.pdf]

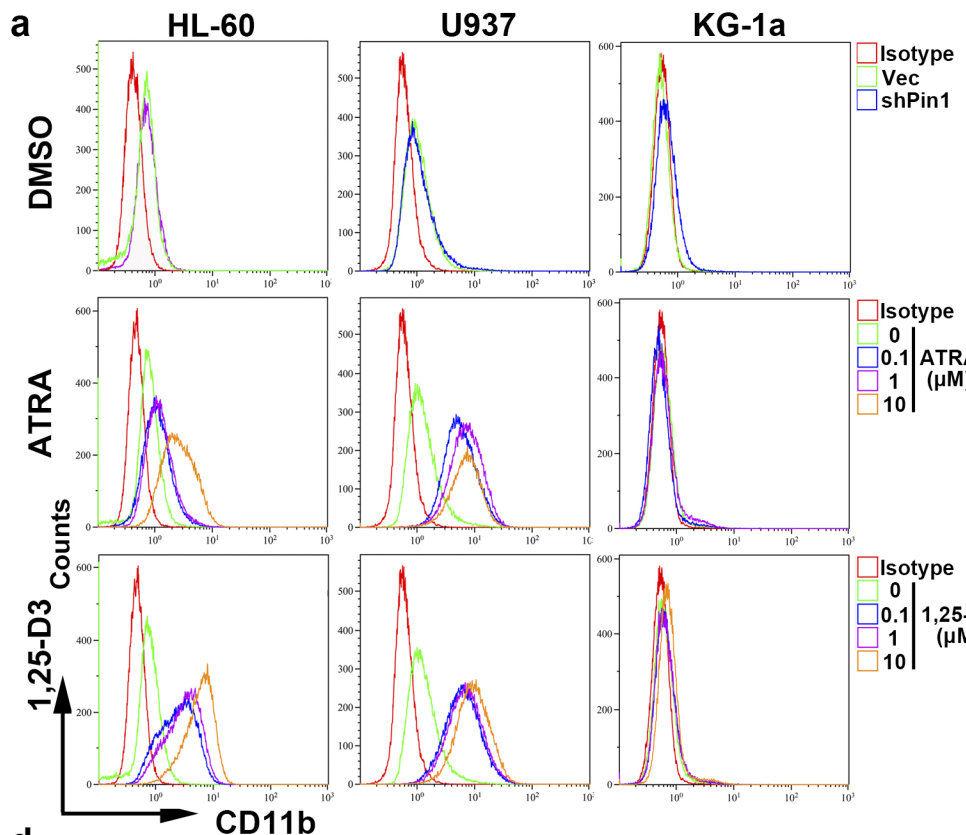

**b**

| treatment    | HL-60      | U937 | KG-1a |
|--------------|------------|------|-------|
|              | CD11b+ (%) |      |       |
| Isotype      | 0.2        | 0.1  | 0.5   |
| Vec          | 5.1        | 4.0  | 0.6   |
| shPin1       | 7.6        | 7.9  | 1.5   |
| 0            | 10.5       | 10.0 | 1.8   |
| ATRA (μM)    | 0.1        | 36.8 | 88.7  |
| 1            | 43.1       | 93.1 | 2.1   |
| 10           | 86.6       | 92.8 | 4.0   |
| 0            | 10.9       | 10.4 | 1.7   |
| 1,25-D3 (μM) | 0.1        | 78.2 | 87.8  |
| 1            | 86.4       | 90.7 | 2.6   |
| 10           | 98.3       | 96.5 | 3.7   |

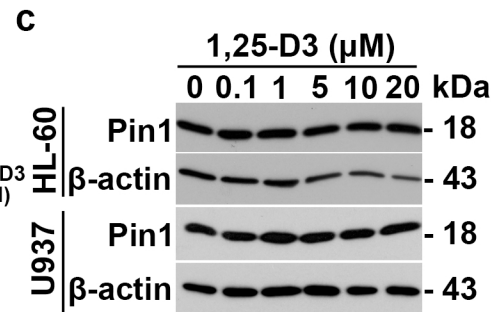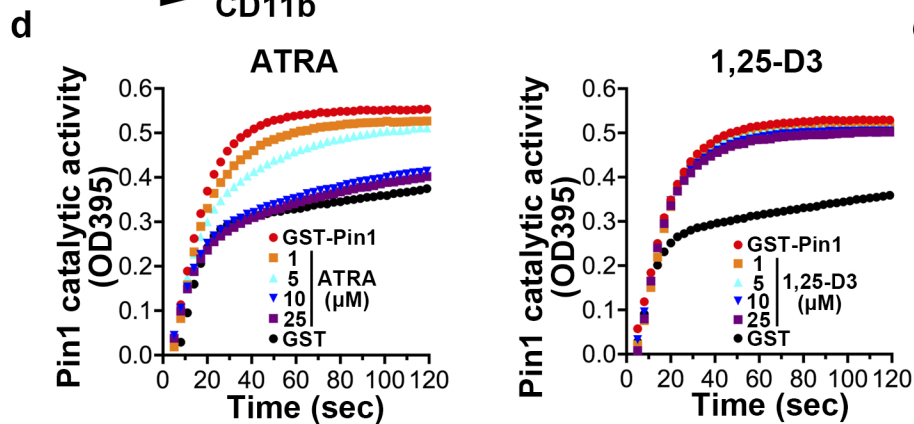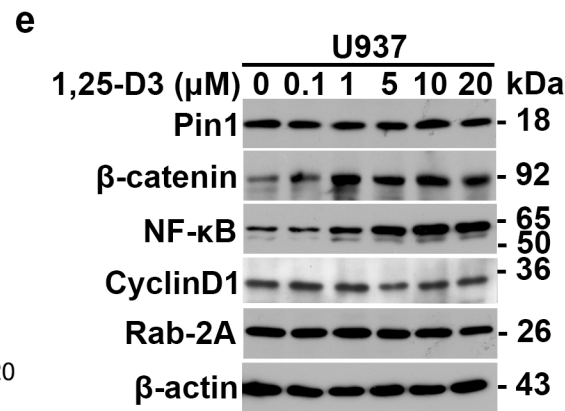

Supplementary Figure 3

Supplement: Supplementary file 3 — Figure S3. 1,25-(OH)2VitaminD3, does not affect Pin1 stability and function. a, b The expression of CD11b were assayed by FACS in HL-60, U937 and KG-1a at 72 h after treatment (a). 1, 25-D3 induces HL-60 and U937 differentiation, but not KG-1a. The differentiation state of each cell was assayed by the percentages of CD11b positive cells in indicated cell lines (b). c Pin1 protein levels were not changed after 72 h incubation of 1, 25-D3 in HL-60 and U937. d 1,25-D3 does not inhibit PPIase activity of Pin1. Pin1 was incubated with different concentrations of 1, 25-D3, followed by chymotrypsin-coupled PPIase assay. e Pin1 downstream oncoproteins were assayed after 72 h incubation of 1,25-D3 in U937. (PDF 2712 kb) [file 13045_2018_611_MOESM3_ESM.pdf]

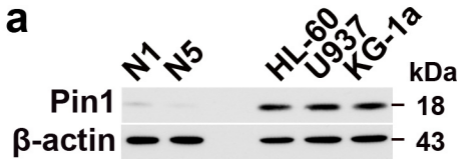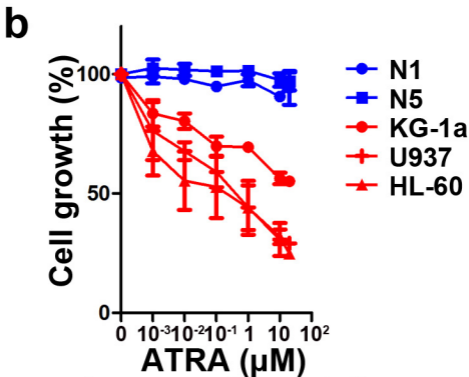

**Supplementary Figure 4**

Supplement: Supplementary file 4 — Figure S4. Immortalized normal blood cells were resistant to ATRA. a Pin1 protein levels in two immortalized normal blood cells (N1 and N5 cells) were assayed by immunoblotting and compared with AML cell lines (HL-60, U937 and KG-1a). N was indicated normal blood cells. b After 3 days treatment of different concentrations of ATRA, cell growth rates were determined by CellTiter-Glo® 2.0 Assay. N1 and N5 cells were completely resistant to ATRA, compared with leukemia cell lines. (PDF 222 kb) [file 13045_2018_611_MOESM4_ESM.pdf]
